# Tea’s Characteristic Components Eliminate Acrylamide in the Maillard Model System

**DOI:** 10.3390/foods13172836

**Published:** 2024-09-06

**Authors:** Zhihao Ye, Haojie Xu, Yingying Xie, Ziqi Peng, Hongfang Li, Ruyan Hou, Huimei Cai, Wei Song, Chuanyi Peng, Daxiang Li

**Affiliations:** 1 State Key Laboratory of Tea Plant Biology and Utilization, Anhui Agricultural University, Hefei 230036, China; yzh971014@163.com (Z.Y.); 18439465028@163.com (H.X.); xieyy1029@163.com (Y.X.); 15156513821@163.com (Z.P.); lihongfang@ahau.edu.cn (H.L.); hry@ahau.edu.cn (R.H.); chm@ahau.edu.cn (H.C.); song86@126.com (W.S.); dxli@ahau.edu.cn (D.L.); 2 Key Laboratory of Food Nutrition and Safety, Anhui Agricultural University, Hefei 230036, China; 3 Anhui Provincial Key Laboratory of Food Safety Monitoring and Quality Control, Hefei 230036, China

**Keywords:** acrylamide, tea’s characteristic components, Maillard model, elimination effect, possible mechanism

## Abstract

This study investigated the effects of various characteristic components of tea—theaflavins, catechins, thearubigins, theasinensins, theanine, catechin (C), catechin gallate (CG), epicatechin (EC), epicatechin gallate (ECG), epigallocatechin (EGC), epigallocatechin gallate (EGCG), gallocatechin (GC), and gallocatechin gallate (GCG)—on acrylamide formation. The results revealed that most of tea’s characteristic components could significantly eliminate acrylamide, ranked from highest to lowest as follows: GC (55.73%) > EC (46.31%) > theaflavins (44.91%) > CG (40.73%) > thearubigins (37.36%) > ECG (37.03%) > EGCG (27.37%) > theabrownine (22.54%) > GCG (16.21%) > catechins (10.14%) > C (7.48%). Synergistic elimination effects were observed with thearubigins + EC + GC + CG, thearubigins + EC + CG, thearubigins + EC + GC, theaflavins + GC + CG, and thearubigins + theaflavins, with the reduction rates being 73.99%, 72.67%, 67.62%, 71.03%, and 65.74%, respectively. Tea’s components reduced the numbers of persistent free radicals to prevent acrylamide formation in the model system. The results provide a theoretical basis for the development of low-acrylamide foods and the application of tea resources in the food industry.

## 1. Introduction

In the thermal processing of foods, acrylamide is a harmful substance that forms during the Maillard reaction between reducing sugars and asparagine [1]. Acrylamide is not naturally present in raw foods, but usually forms during cooking at a high temperature (over 120 °C), especially when starchy and carbohydrate-rich foods are cooked [2]. In 1994, acrylamide was classified as a level 2A carcinogen by the International Agency for Research on Cancer [3], which caused global concern regarding the substance. In 2015, the European Food Safety Authority provided health-based guidance regarding the maximum permissible levels of acrylamide in foods [4], and in 2017, the European Union issued regulations specifying benchmark levels of acrylamide [5]. For example, coffee and french fries have high acrylamide levels, at 389 and 1499 μg/kg, and their recommended benchmark levels are 750 and 400 μg/kg, respectively.

An extensive research effort has been expended in attempts to minimize the amount of acrylamide that forms during food processing. The first step is assessing raw materials. Different varieties of crops, especially potatoes and cereals, contain differing levels of reducing sugars and asparagine [6], which are the precursor substances for the synthesis of acrylamide. Thus, the cultivation and selection of raw materials with low levels of sugar and asparagine play a crucial role in reducing the degree of acrylamide formation [3,7]. Second, several processing conditions—including temperature, time, and pH—strongly influence the likelihood of acrylamide formation during cooking [8,9]. Modifying these parameters can lead to major reductions in acrylamide levels. For instance, lowering the cooking temperature [10,11], extending the cooking time [11], and adjusting the pH toward acidity [12,13] have all been reported to reduce acrylamide levels in food products. Enzyme technology has also shown potential for acrylamide elimination [14,15]. Furthermore, the application of food additives, a simple strategy that can be easily implemented, can inhibit acrylamide formation without requiring a change in the raw materials or processing technologies that are used. Zamora et al. [11] found that dipalmitoylphosphatidylethanolamine reduced the amount of acrylamide produced in the glucose–asparagine (Glc-Asn) model system and that the inhibitory effect was significantly correlated with the concentration of amino phospholipid [11]. In another study, mercaptan could react with acrylamide to form stable adducts, whereas under aerobic conditions, no additional products were identified, but acrylamide formation was noticeably less severe [16]. 

Many plants’ or extracts’ pronounced antioxidant properties are known to naturally inhibit the formation of acrylamide. Liu et al. found that soaking french fries in a 1.34% sodium alginate solution reduced acrylamide production by 76.59% and had no effect on their sensory properties; this soaking resulted in the formation of a protective layer on the surface of the fries [17]. In a model system, naringin was found to inhibit acrylamide formation at a rate of 20–50%; high-performance liquid chromatography (HPLC)–tandem mass spectrometry (MS/MS) and nuclear magnetic resonance analyses revealed that this occurred because naringin reacted with the precursors of acrylamide [18]. Trujillo-mayol et al. [19] added 0.5% avocado peel extract to beef and vegetarian burgers to reduce the levels of acrylamide and heterocyclic amines in the cooked burgers [19]. In addition, a significant reduction in acrylamide was achieved by soaking chicken legs and wings in green tea extract before they were fried [20]; this reduction was attributed to an interaction between the precursor substances of acrylamide and the components of the green tea extract, including catechin and gallic acid. Compared with synthetic antioxidants such as butylated hydroxyanisole and butylated hydroxytoluene, plant polyphenols are more acceptable for consumers.

Plant polyphenols, which are phytochemicals, have attracted a great deal of attention because they are natural antioxidants. Tea is a polyphenol-rich food, and tea polyphenols have multiple pharmacological and physiological functionalities due to their well-known antioxidant attributes. China’s overall tea production in 2023 was 3.55 million tons [21]; the production of summer and autumn teas accounts for more than 50% of Chinese tea production, but the rate of utilization of these types of tea is low because they are bitter and astringent in taste and have poor aromatic qualities. However, the leaves of these teas, which contain numerous polyphenols, have considerable potential for use in the elimination of acrylamide. In the current study, the abilities of the aforementioned teas’ characteristic components—theanine, tea polyphenols and the products of their different degrees of oxidation (theaflavins, thearubigins, and theasinensins), and catechins—to eliminate acrylamide were systematically evaluated, and the possible mechanism underlying their ability to reduce acrylamide was also explored by monitoring the scavenging of free radicals.

## 2. Materials and Methods

### 2.1. Reagents and Materials

Acrylamide (C_3_H_5_NO, ≥99.90%) and ^13^C_3_–acrylamide were purchased from Dr. Ehrenstorfer (Augsburg, Germany) and Beijing Manhage Biotechnology (Beijing, China), respectively. The asparagine, glucose, Na_2_HPO_4_, and K_2_HPO_4_ used in this study were analytical grade and obtained from Merk (Darmstadt, Germany). N-Propylethylenediamine (PSA) was purchased from Shanghai Anpel Laboratory Technologies (Shanghai, China), and deionized water (18.2 MΩ cm) was prepared using a Milli-Q Gradient system (Billerica, MA, USA). HPLC grade characteristic components of tea—catechin (C), catechin gallate (CG), epicatechin (EC), epicatechin gallate (ECG), epigallocatechin (EGC), epigallocatechin gallate (EGCG), gallocatechin (GC), gallocatechin gallate (GCG), tea polyphenols, theaflavins, thearubigins, theabrownine, and theanine—were purchased from Shanghai Yuanye Biotechnology (Shanghai, China). Instant green tea was purchased from Zhejiang Minghuang Natural Products Development.

### 2.2. Glc-Asn Thermal Model Reaction

The Maillard model reaction was conducted using a previously described method with some minor modifications [22]. In brief, an equimolar solution of glucose and asparagine was accurately prepared in phosphate buffer (0.1 M, pH 6.86), and 4 mL of the solution was transferred to a 25 mL thick-walled pressurized glass tube in an oil bath (DF-1015, Shanghai Lichen Instrument Technology, Shanghai, China) at 180 °C and kept for 30 min. The sample was then allowed to cool to room temperature, and 0.1 mL of ^13^C_3_–acrylamide standard solution (10 mg/L) was added. Subsequently, the total volume was adjusted to 100 mL by adding deionized water, and 2 mL was collected and mixed with 0.2 g of PSA. The mixture was vortexed for 2 min and then centrifuged at 8000 rpm for 5 min at 25 °C. After centrifugation, the supernatant was collected and filtered through a 0.22 μm cellulose syringe filter before undergoing liquid chromatography (LC)–mass spectrometry analysis.

### 2.3. Elimination Evaluation of Effects of Tea’s Characteristic Components on Acrylamide in Maillard Model System

In the Maillard model system, the effects of the following substances on acrylamide levels were evaluated: instant green tea (0.01, 0.1, 1, 10, and 100 g/mol Asn), tea polyphenols (0.1, 0.25, 0.5, 1, and 2 g/mol Asn), theanine (0.1, 0.25, 0.5, 1, and 2 g/mol Asn), C (0.025, 0.05, 0.1, 0.25, and 0.5 g/mol Asn), EC (0.025, 0.05, 0.1, 0.25, and 0.5 g/mol Asn), EGC (0.025, 0.05, 0.1, 0.25, and 0.5 g/mol Asn), GC (0.05, 0.1, 0.25, 0.5, and 1 g/mol Asn), CG (0.025, 0.05, 0.1, 0.25, and 0.5 g/mol Asn), ECG (0.025, 0.05, 0.1, 0.25, and 0.5 g/mol Asn), EGCG (0.025, 0.05, 0.1, 0.25, and 0.5 g/mol Asn), GCG (0.05, 0.1, 0.25, 0.5, and 1 g/mol Asn), theaflavins (0.05, 0.1, 0.25, 0.5, and 1 g/mol Asn), thearubigins (0.05, 0.1, 0.25, 0.5, and 1 g/mol Asn), and theabrownine (0.05, 0.1, 0.25, 0.5, and 1 g/mol Asn). The optimal composition with equal weights of well-behaved characteristic components of tea was then determined using a comprehensive experimental design.

### 2.4. LC–Triple Quadrupole (QQQ) MS/MS Analysis of Acrylamide in the Maillard Model System

An LC-QQQ-MS/MS device equipped with an electrospray ionization source (AB SCIEX, Boston, MA, USA) coupled with a SCIEX high-performance liquid spectrometer system was used to determine the levels of acrylamide that formed in the Maillard model system [23]. The analyte was separated on a Waters Acquity UPLC BEH Shield RP18 column (1.7 μm, 1.0 mm × 50 mm, Waters, MA, USA) at 40 °C under a flow rate of 0.2 mL/min. The injection volume was 2 μL, and during isocratic elution, the mobile phase consisted of methanol/0.1% formic acid in deionized water (10:90, *v/v*). Acrylamide was identified and quantified using the multiple reaction monitoring mode; the transitions *m/z* 72→55 and *m/z* 72→27 indicated acrylamide, whereas *m/z* 75→58 and *m/z* 75→29 indicated ^13^C_3_–acrylamide.

### 2.5. Free Radicals in the Maillard Model System

Electron paramagnetic resonance (EPR) was performed to investigate the variation in the free radicals in the Glc-Asn model system before and after the addition of the characteristic components of tea and thereby explore the possible mechanism of acrylamide reduction. The EPR process was as follows: A reacted sample was collected and prepared as a 1 mg/mL solution, and equal volumes of this solution and a radical scavenger solution were mixed. Subsequently, a capillary was employed to draw an appropriate amount of the mixed solution into a quartz tube, and the total spin number was recorded on a Bruker EMX plus 6/1 spectrometer (Billerica, Massachusetts, USA) equipped with an Oxford Instrument. The instrument parameters were as follows: center magnetic field, 3480 G; scan time and width, 60 s and 50 G, respectively; microwave power, 6 mW; and modulation amplitude, 3.0 G.

### 2.6. Statistical Analysis

All experiments were performed at least in triplicate, and the data were statistically analyzed using IBM SPSS software (version 26.0, SPSS, Chicago, IL, USA). The results are presented as means ± standard deviations (SDs). Analysis of variance and the least significant difference test or Dunnett’s test, selected on the basis of the results of Bartlett’s test for equal variances, were used to determine the differences between means. Significance was considered at *p* < 0.05.

## 3. Results and Discussion

### 3.1. The Effects of Instant Green Tea on Acrylamide Levels in the Glc-Asn Model System

**Figure 1:**
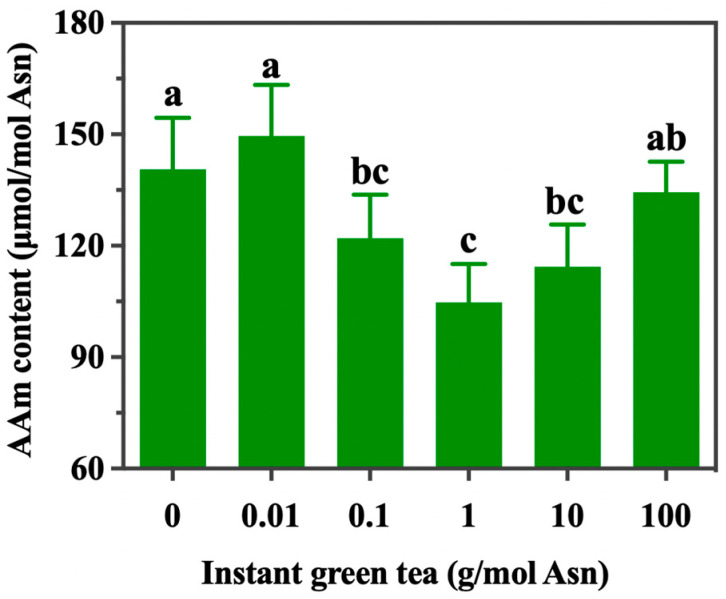
The effects of instant green tea on acrylamide reduction in the Glc-Asn model system. The different letters indicate significant differences at *p* < 0.05 at different instant green tea concentrations.

The effects of instant green tea on the profile of acrylamide in the Glc-Asn model system are illustrated in Figure 1. The level of acrylamide under the control condition (i.e., 0 g/mol Asn) was 140.58 ± 13.92 μmol/mol Asn. When the amounts of instant green tea were 0.1, 1, and 10 g/mol Asn, the levels of acrylamide in the model system were significantly lower; the rates of inhibition were 13.22–25.48%. However, the low dose (<0.1 g/mol Asn) and high dose (>10 g/mol Asn) of instant green tea did not have significantly inhibitive effects. Morales et al., Li et al., and Budryn et al. have reported that the addition of an aqueous extract of green tea or tea polyphenols could significantly reduce the levels of acrylamide in fried potatoes, bread, and fried yeast donuts, with maximum reductions of 62%, 43%, and 15%, respectively [24,25,26]. However, another study discovered that the addition of a high amount of green tea extract to fried yeast donuts enhanced the formation of acrylamide [26].

Tea can be classified into six basic types on the basis of its degree of fermentation with polyphenol oxidase and the microorganisms of tea polyphenols: green tea, white tea, yellow tea, oolong tea, black tea, and brick tea [27]. Green tea has the highest concentration of total catechins and the strongest antioxidant activity when it is prepared from the dried leaves of a single *Camellia sinensis* cultivar [28]. Green tea contains abundant secondary metabolites, including tea polyphenols and theanine; however, the specific characteristic component of green tea that contributes to acrylamide reduction remains unclear, as do the possible synergistic or antagonistic effects.

### 3.2. The Effects of Tea’s Characteristic Components on Acrylamide Levels in the Glc-Asn Model System

**Figure 2:**
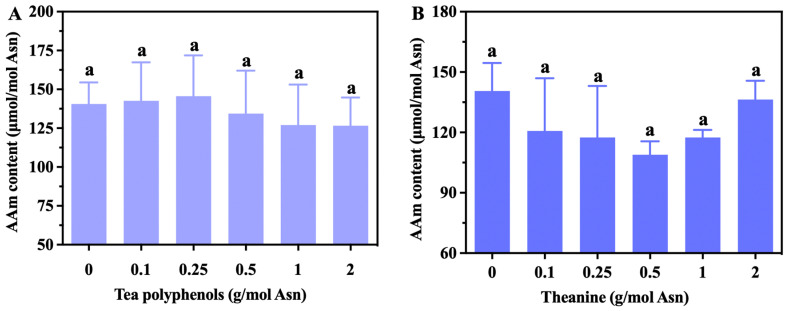
The effects of tea polyphenols (**A**) and theanine (**B**) on acrylamide reduction in the Glc-Asn model system. The different letters indicate significant differences at *p* < 0.05 at different concentrations.

To better understand the effects of tea’s specific components on acrylamide levels, different characteristic components of tea—tea polyphenols, theanine, and catechins—were investigated in the Glc-Asn model system. As illustrated in Figure 2A,B, neither tea polyphenols nor theanine (when applied at levels of 0.1, 0.25, 0.5, 1, and 2 g/mol Asn) significantly affected acrylamide formation (*p* < 0.05). According to the performance results for instant green tea, tea polyphenols, and theanine, these substances had clear effects antagonistic to acrylamide reduction. Tea polyphenols, also called tea tannings or tea tannins, mainly comprise catechins, flavonoids, anthocyanins, and phenolic acids, with the content of catechin compounds being the highest; catechin compounds account for 60–80% of the total amount of tea polyphenols. Dozens of studies have reported that plant polyphenols with different structures have differing impacts on the formation of acrylamide [1]. Apple extract has been reported to inhibit acrylamide formation, and dragon fruit and hesperetin have been reported to enhance acrylamide formation [29,30]. Reactive carbonyl groups of plant polyphenols were reported by one study to be the major sites of acrylamide formation [1]. In the present study, eight catechin monomers were individually investigated to evaluate their roles in acrylamide elimination.

**Figure 3:**
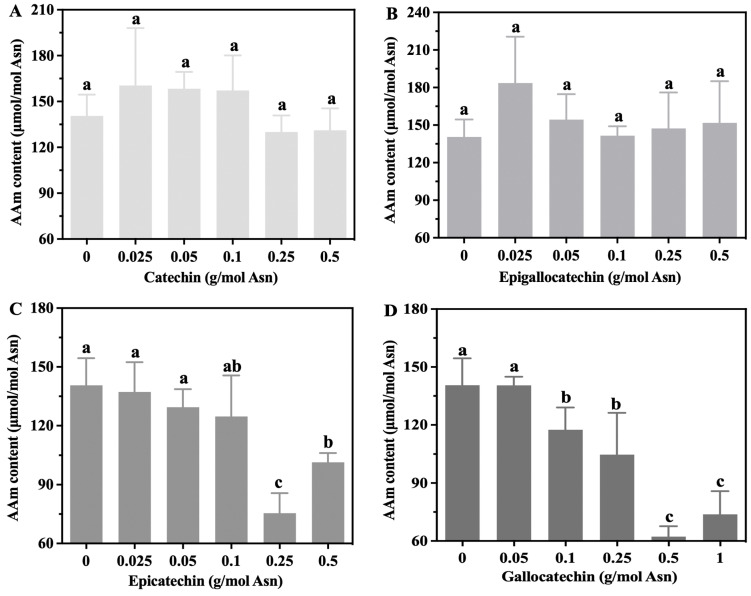

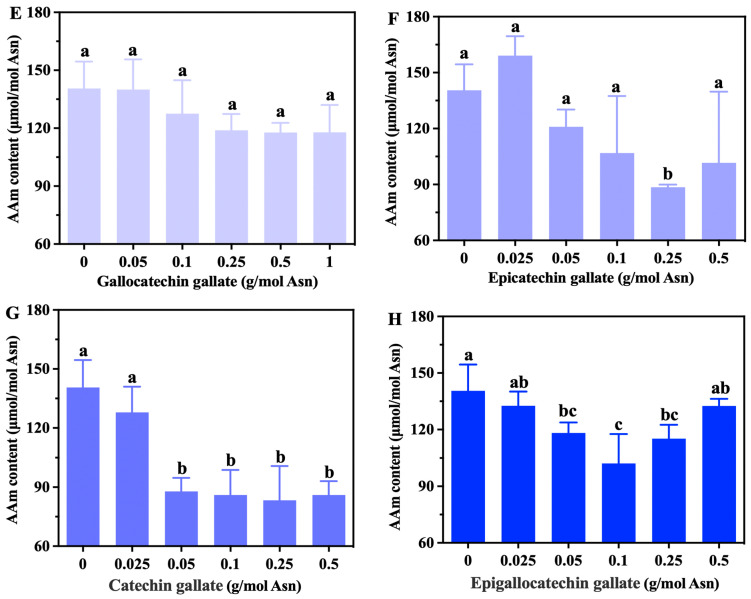
The effects of C (**A**), EGC (**B**), EC (**C**), GC (**D**), GCG (**E**), ECG (**F**), CG (**G**), and EGCG (**H**) on acrylamide reduction in the Glc-Asn model system. The different letters indicate significant differences at *p* < 0.05 at different concentrations.

The effects of nonester catechin monomers (C, EC, EGC, and GC) on acrylamide levels in the Glc-Asn model system are presented in Figure 3A–D. C (applied at levels of 0.025–0.5 g/mol Asn) and EGC (0.025–0.5 g/mol Asn) did not have an inhibitory effect (*p* > 0.05). By contrast, the addition of EC (0.25–0.5 g/mol Asn) and GC (0.1–1 g/mol Asn) resulted in significantly lower acrylamide content; the highest level of inhibition for EC was found to be 46.31%, achieved at 0.25 g/mol Asn, and that for GC was discovered to be 55.73%, achieved at 0.5 g/mol Asn. Figure 3E–H present the inhibition performances for ester catechin monomers (GCG, ECG, CG, and EGCG). The effect of GCG was not sufficient to achieve a significant reduction in acrylamide levels (*p* > 0.05, Figure 3E). The additions of ECG, CG, and EGCG resulted in less acrylamide when they were applied at the levels of 0.25, 0.05–0.5, and 0.05–0.25 g/mol Asn, respectively; the maximum inhibitions were 37.03%, 40.73%, and 27.37%, respectively. The effects of these catechin monomers were discovered to not be dose-dependent, implying that the inhibition of acrylamide formation may be dependent on the structure and concentration of the specific polyphenol. EC and EGCG were reported to terminate the formation of Maillard products in the model system and in UHT milk during storage [30]. Hedegaard et al. [31] discovered that 1.0 mM EC or EGCG decreased the acrylamide content in a model food system, whereas the lower concentration of 0.1 mM did not. Remarkably, numerous studies have obtained evidence indicating that the correlation between acrylamide formation and the concentration of plant polyphenols is nonlinear [1,32]. For example, a phenolic extract from virgin olive oil was found to efficiently inhibit acrylamide formation in the Glc-Asn model system, whereas the opposite effect occurred when a higher concentration of the phenolic extract was employed [33] and when apple proanthocyanidins were applied to fried potato [34]. The link between antioxidant capacity and elimination effects thus remains unclear. Plant polyphenols’ prevention of the formation of acrylamide may be attributable to the reaction of these polyphenols with acrylamide, acrylamide precursors, or intermediates during thermal processing.

### 3.3. The Effects of Tea Polyphenol Oxides on Acrylamide Levels in the Glc-Asn Model System

**Figure 4:**
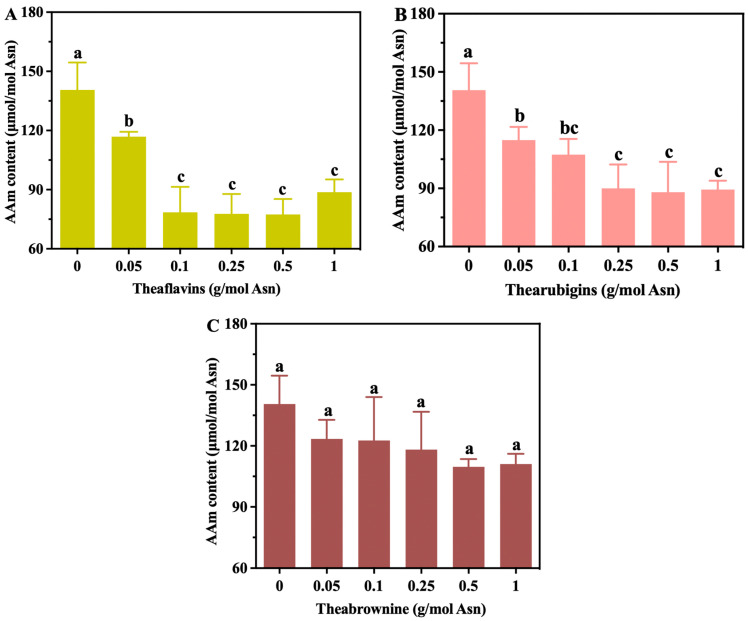
Effects of theaflavins (**A**), thearubigins (**B**), and theabrownine (**C**) on acrylamide reduction. The different letters indicate significant differences at *p* < 0.05 at different concentrations.

Tea polyphenols have multiple phenolic hydroxyl groups and are easily oxidized into theaflavins and thearubigins by polyphenol oxidase; they can also be converted into theabrownine with the assistance of microorganism fermentation. In the current study, the ability of tea polyphenol oxides to inhibit acrylamide in the model system was assessed (Figure 4). The results revealed that oxidation products of tea polyphenols (theaflavins and thearubigins) had strong inhibitory effects on acrylamide, with maximum inhibitions of 44.91% and 37.36%, respectively, when applied at 0.5 g/mol Asn. Thus, minor oxidation can improve tea polyphenols’ ability to reduce the amount of acrylamide (Figure 2A). However, theabrownine did not have an inhibitory effect (*p* > 0.05, Figure 4C).

### 3.4. Synergistic or Antagonistic Effects of Tea’s Characteristic Components on Acrylamide Formation

To investigate the possible synergistic or antagonistic effects of tea’s characteristic components on acrylamide levels, five characteristic components of tea with inhibition rates higher than 35% (theaflavins, thearubigins, EC, GC, and CG) were selected and grouped in 26 combinations. Combinations with component levels of 1 g/mol Asn in equal masses were added to the Glc-Asn model system separately, and the acrylamide content in the systems with these combinations was determined; the results are presented in Table 1. Both synergistic and antagonistic effects were discovered for certain combinations. Combinations 3 (theaflavins + thearubigins + EC + CG), 4 (theaflavins + thearubigins + GC + CG), 5 (theaflavins + EC + GC + CG), 6 (thearubigins + EC + GC + CG), 7 (EC + GC + CG), 9 (thearubigins + EC + CG), 10 (thearubigins + EC + GC), 11 (theaflavins + GC + CG), and 26 (theaflavins + thearubigins) were discovered to have a synergistic effect, that is, to result in much lower acrylamide levels than the individual components did. The acrylamide reduction rates of combinations 1 (theaflavins + thearubigins + EC + GC + CG), 12 (theaflavins + EC + CG), 16 (theaflavins + thearubigins + EC), and 22 (thearubigins + EC) were only 19.52%, 10.65%, 28.19%, and 21.54%, respectively. These combinations were thus concluded to have clear antagonistic effects. However, the mechanisms underlying the synergistic and antagonistic effects of these components remain unclear.

The elimination rates obtained in this study were compared with those previously reported. Compared with the rates for other plant polyphenols, the elimination rates of the tea characteristic components considered in the present study were favorable for their use for the elimination of acrylamide (Table 2). This provides an effective option for the high-value utilization of tea resources, especially summer and autumn tea resources.

### 3.5. The Effects of Tea’s Characteristic Components on Free Radical Generation in the Glc-Asn Model System

EPR is the most straightforward and practical method for detecting free radicals. In EPR, different spectral peaks occur when different scavengers are added to samples, and the nature of the free radicals that are present can be determined by their spectral characteristics. Figure S1 presents the EPR spectra of the persistent radicals in the Glc-Asn model system before and after the addition of tea’s characteristic components. The amplitude of the spectra obtained after this addition is smaller than that of the spectra obtained before the addition. The total number of spins before addition was 1.37 × 10^14^; after EC, CG, GC, and thearubigins were added, the total numbers of spins were 5.60 × 10^13^, 3.93 × 10^13^, 4.46 × 10^13^, and 5.21 × 10^13^, respectively. This finding suggests that the mechanism through which acrylamide formation is prevented by tea’s characteristic components may involve the prevention of the formation of persistent free radicals. The protection mechanism of plant polyphenols is likely strongly related to the scavenging of free radicals from the Maillard reaction [26,38]. Plant polyphenols are a large group of naturally occurring polyphenolic hydroxyl groups, the hydrogen atoms of which can scavenge free radicals and terminate the propagation of free radical chain reactions [1].

## 4. Conclusions

The comprehensive influence of tea and its characteristic components on the formation of acrylamide in the Glc-Asn model system was investigated. Within the selected ranges of addition levels, all characteristic components of tea, with the exception of EGC, were discovered to negatively affect the formation of acrylamide in the model system. The components, ranked from highest to lowest rate, had the following acrylamide reduction rates: GC (55.73%) > EC (46.31%) > theaflavins (44.91%) > CG (40.73%) > thearubigins (37.36%) > ECG (37.03%) > EGCG (27.37%) > green tea (25.48%) > theanine (22.54%) > GCG (16.21%) > catechins (10.14%) > C (7.48%). Combinations 7 (EC + GC + CG), 9 (thearubigins + EC + CG), 10 (thearubigins + EC + GC), 11 (theaflavins + GC + CG), and 26 (theaflavins + thearubigins) were found to have favorable negative effects on acrylamide. These combinations achieved acrylamide reduction rates higher than 65% and rates that were 11.89–36.63% higher than those achieved for the individual components. Tea’s characteristic components reduce the amount of acrylamide that forms by preventing the formation of persistent free radicals. This study provides a practical strategy and useful guidelines for controlling the amounts of acrylamide in thermally processed foods using tea and its characteristic components as food ingredients.

## Figures and Tables

**Table 1:** Effects of 26 complexes on acrylamide formation in Glc-Asn model system.

Combination	Ingredient					AAm Content (μmol/mol Asn)	Reduction Rate (%)
	Theaflavins	Thearubigins	EC	GC	CG		
Control	-	-	-	-	-	140.58 ± 13.92 ^a^	ND
1	+	+	+	+	+	113.14 ± 10.80 ^bc^	19.52
2	+	+	+	+	-	74.39 ± 14.61 ^ghij^	47.08
3	+	+	+	-	+	55.97 ± 19.74 ^jkl^	60.19
4	+	+	-	+	+	51.14 ± 5.22 ^kl^	63.62
5	+	-	+	+	+	61.66 ± 3.35 ^ijk^	56.14
6	-	+	+	+	+	36.56 ± 1.79 ^l^	73.99
7	-	-	+	+	+	50.28 ± 3.93 ^kl^	64.24
8	-	+	-	+	+	77.78 ± 13.13 ^fghi^	44.67
9	-	+	+	-	+	38.42 ± 3.71 ^l^	72.67
10	-	+	+	+	-	45.53 ± 3.64 ^kl^	67.62
11	+	-	-	+	+	40.73 ± 2.34 ^l^	71.03
12	+	-	+	-	+	125.61 ± 17.96 ^ab^	10.65
13	+	-	+	+	-	82.79 ± 2.70 ^efgh^	41.11
14	+	+	-	-	+	96.47 ± 5.59 ^cdef^	31.38
15	+	+	-	+	-	96.75 ± 4.03 ^cdef^	31.18
16	+	+	+	-	-	100.96 ± 14.17 ^cde^	28.19
17	-	-	-	+	+	73.94 ± 0.96 ^hij^	47.40
18	-	-	+	-	+	91.08 ± 3.86 ^defg^	35.21
19	-	-	+	+	-	72.32 ± 1.99 ^ghij^	48.56
20	-	+	-	-	+	72.65 ± 13.71 ^ghij^	48.32
21	-	+	-	+	-	70.87 ± 11.83 ^hij^	49.59
22	-	+	+	-	-	110.30 ± 24.53 ^bcd^	21.54
23	+	-	-	-	+	92.51 ± 9.22 ^defg^	34.19
24	+	-	-	+	-	91.70 ± 11.91 ^defgh^	34.77
25	+	-	+	-	-	86.39 ± 17.54 ^efgh^	38.55
26	+	+	-	-	-	48.165 ± 5.3 ^kl^	65.74

Note: The data presented are the means and standard deviations of three samples (n = 3). Lowercase letters indicate significant differences (*p* < 0.05); “+” indicates added, “-” indicates not added, and “ND”indicates observations with no detection.

**Table 2:** A comparison of the proposed tea characteristic components and the reported plant polyphenols for AAm elimination.

Plant Polyphenols	Matrix	Amount	Elimination Rate (%)	Reference
Tea characteristic components	Glc-Asn model system	0.25–0.5 g/mol Asn	37.36–55.73	This study
Combinations		1 g/mol Asn	65.74	
			67.62	
			71.03	
			72.67	
			73.99	
Naringin		11.6 mg/mol Asn	20	[18]
		22.3 mg/mol Asn	40	
		58.1 mg/mol Asn	60	
Apigenin		18.9 g/mol Asn	67.17	[35]
Cyanidenon		20.02 g/mol Asn	84.17	
Quercetin		2.11 g/mol Asn	80.11	
Glycyrrhizin		0.18 g/mol Asn	88.77	
Liquiritin		0.29 g/mol Asn	81.65	
Genistein		1.89 g/mol Asn	86.51	
Silymarin		3.37 mg/mol Asn	83.99	
Garlic powder (freeze-dry)		41.67 g/mol Asn	41	[36]
Garlic powder (oven-dry)			37.3	
Garlicin		9.1 mg/mol Asn	71.3	
Antioxidant of bamboo leaves	Potato chips	0.1% (*w*/*w*)	74.1	[37]
	French fries	0.01% (*w*/*w*)	76.1	
	Chinese fried dough stick	0.1% (*w*/*w*)	82.9	
	Fried chicken wings	0.5% (*w*/*w*)	59	

## Data Availability

The original contributions presented in the study are included in the article/Supplementary Material, further inquiries can be directed to the corresponding author.

## Supplementary Materials

The following supporting information can be downloaded at: https://www.mdpi.com/article/10.3390/foods13172836/s1, Figure S1: Effects of EC, CG, GC, and thearubigins on free radicals in model system.
