# Supplementary material for: Tea’s Characteristic Components Eliminate Acrylamide in the Maillard Model System
Source: Foods. 2024 Sep 6;13(17):2836. doi: 10.3390/foods13172836 (PMC11395303; doi:10.3390/foods13172836)
Supplement: Supplementary file 1 [file foods-13-02836-s001.zip › foods-3106810-supplementary.pdf]

Tea's Characteristic Components Eliminate Acrylamide in the Maillard Model System

Zhihao Ye [1,2,3,†], Haojie Xu [1,2,3,†], Yingying Xie [1,2,3], Ziqi Peng [1,2,3], Hongfang Li [1,2,3], Ruyan Hou [1,2,3], Huimei Cai [1,2,3], Wei Song [3], Chuanyi Peng [1,2,3,*] and Daxiang Li [1]

[1] State Key Laboratory of Tea Plant Biology and Utilization, Anhui Agricultural University,
Hefei 230036, China; yzh971014@163.com (Z.Y.); 18439465028@163.com (H.X.); xieyy1029@163.com (Y.X.); 15156513821@163.com (Z.P.); lihongfang@ahau.edu.cn (H.L.); hry@ahau.edu.cn (R.H.); chm@ahau.edu.cn (H.C.); song86@126.com (W.S.); dxli@ahau.edu.cn (D.L.)
[2] Key Laboratory of Food Nutrition and Safety, Anhui Agricultural University, Hefei 230036, China
[3] Anhui Provincial Key Laboratory of Food Safety Monitoring and Quality Control, Hefei 230036, China
* Correspondence: pcy0917@ahau.edu.cn or pcy72988@126.com; Tel./Fax: +86-0551-65786421
† These authors contributed equally to this work.

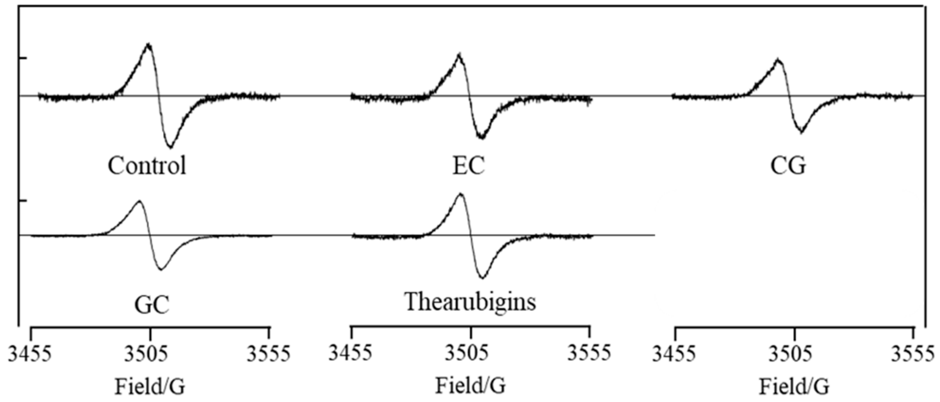

Figure S1 Effects of EC, CG, GC, and thearubigins on free radicals in model system
